# A numerical technique for linear elliptic partial differential equations in polygonal domains

**DOI:** 10.1098/rspa.2014.0747

**Published:** 2015-03-08

**Authors:** P. Hashemzadeh, A. S. Fokas, S. A. Smitheman

**Affiliations:** 1 Department of Applied Mathematics and Theoretical Physics, University of Cambridge, Cambridge CB3 0WA, UK; 2 Research Center in Mathematics, Academy of Athens, Athens 11527, Greece

**Keywords:** elliptic partial differential equation, Laplace, Helmholtz equation, modified Helmholtz equation, Fokas transform, Fokas method

## Abstract

Integral representations for the solution of linear elliptic partial differential equations (PDEs) can be obtained using Green's theorem. However, these representations involve both the Dirichlet and the Neumann values on the boundary, and for a well-posed boundary-value problem (BVPs) one of these functions is unknown. A new transform method for solving BVPs for linear and integrable nonlinear PDEs usually referred to as the *unified transform* (*or the Fokas transform*) was introduced by the second author in the late Nineties. For linear elliptic PDEs, this method can be considered as the analogue of Green's function approach but now it is formulated in the complex Fourier plane instead of the physical plane. It employs two *global relations* also formulated in the Fourier plane which couple the Dirichlet and the Neumann boundary values. These relations can be used to characterize the unknown boundary values in terms of the given boundary data, yielding an elegant approach for determining the *Dirichlet to Neumann map*. The numerical implementation of the unified transform can be considered as the counterpart in the Fourier plane of the well-known boundary integral method which is formulated in the physical plane. For this implementation, one must choose (i) a suitable basis for expanding the unknown functions and (ii) an appropriate set of complex values, which we refer to as collocation points, at which to evaluate the global relations. Here, by employing a variety of examples we present simple guidelines of how the above choices can be made. Furthermore, we provide concrete rules for choosing the collocation points so that the condition number of the matrix of the associated linear system remains low.

## 1. Introduction

A new method for analysing boundary-value problems (BVP) for linear and for integrable nonlinear partial differential equations (PDEs) was introduced by the second author in the late Nineties [1–3]. This method, which is usually referred to as the unified transform (or the Fokas transform), has been applied to a variety of linear elliptic PDEs formulated in the interior of a polygon. Important results in this direction include the following: (i) for the Laplace, modified Helmholtz and Helmholtz equations, it is possible to express the solution in terms of integrals in the complex λ-plane (complex Fourier plane). These integrals contain certain integral transforms of the Dirichlet and of the Neumann values on the boundary of the polygon. Hence, these integral formulae provide the analogue of the classical Green's representations, but now the formulation takes place in the complex Fourier plane instead of the physical plane. (ii) The above transforms of the Dirichlet and of the Neumann boundary values are related via two simple *algebraic* equations called *global relations*. These relations provide a characterization of the *generalized Dirichlet to Neumann map*. (iii) By employing the integral representation and global relations mentioned in (i) and (ii), respectively, it has been possible to obtain exact solutions for a variety of problems for which apparently the usual approaches fail, see e.g. [4,5]. (iv) Ashton [6,7] has developed a rigorous approach for deriving well posedness results for linear elliptic PDEs using the new formalism. This includes the analysis of BVPs with distributional data and with corner singularities [8]. (v) The new method can be applied to linear PDEs with *nonlinear* boundary conditions, see e.g. [9–11]. (vi) The first steps have been taken towards extending the unified transform to three dimensions, see e.g. [9,12].

The analysis of the global relations yields a novel numerical technique for the numerical solution of the generalized Dirichlet to Neumann map, i.e. for the determination of the unknown boundary values in terms of the prescribed boundary data [13–21]. Substantial progress in this direction was made by Fornberg and co-worker [14,15]. The global relations couple the finite Fourier transform of the given boundary data with the finite Fourier transform of the unknown boundary values. For the determination of these boundary values one has to (a) choose appropriate basis functions and (b) suitable collocation points in the Fourier plane. For the numerical computation of the finite Fourier transforms of the basis functions, Fornberg and co-worker have used Legendre polynomials, and have employed the fact that the finite Fourier transform of the Legendre polynomials can be expressed in terms of the modified Bessel function of order half integer. In the above-mentioned papers, the authors have also used the so-called Halton nodes for collocation points, and have used the crucial observation that the conditioning of the associated linear system improves if the linear system is *overdetermined*. Here, following Fornberg and co-worker, we also use Legendre polynomials and also overdetermine the relevant system. However, motivated by the results of [21], we introduce a new choice of collocation points. In this way, we are able to improve dramatically the relevant condition number. For example, for a particular BVP considered in [15], the condition number improves from approximately 10^16^ to 4.9.

There exist several different numerical methods available for solving linear elliptic PDEs, which include finite-difference methods, finite-element methods, boundary-integral equations and the method of particular solutions. The main novelty of the method developed in this paper, compared with standard methods, is that it is a boundary-based discretization that does not involve the computation of singular integrals (as opposed to the discretizations of boundary integral equations). Similar ideas have been proposed before in the literature, and the relationship of the method here to these earlier approaches is discussed in detail in §6.

This paper is organized as follows: in §2, we review the concept of *global relations* and obtain these relations for the Laplace, modified Helmholtz and Helmholtz equations in the interior of a polygonal domain. In §3, we approximate the known and unknown boundary values in terms of Legendre polynomials, and derive the *approximate global relations*. In §4, we discuss a convenient choice for collocation points for the particular case of a convex polygon. In §5, we present several numerical calculations comparing the unified transform solution with an exact solution or a numerical solution obtained via the finite-element method. Finally, in §6, we discuss further these results.

## 2. Global relations

For completeness, we first recall the global relations for the Laplace, modified Helmholtz and Helmholtz equations. Solutions of the Laplace equation in the closed domain $\Omega \subset R^{2}$,

2.1 $$u_{xx}+u_{yy}=0,\;(x,y)\in \Omega ,$$

satisfies Green's second identity

2.2 $$\oint_{\partial \Omega }\left(u\frac{\partial v}{\partial N}-v\frac{\partial u}{\partial N}\right)ds=0,$$

where ∂*Ω* denotes the boundary of the domain *Ω*, *v* is a solution of the Laplace equation, and ∂/∂N denotes the derivative along the outward normal direction of the boundary. Letting

2.3 $$z=x+iy,\;\bar{z}=x-iy,$$

noting that $v=e^{-i\lambda z},\lambda \in C^{\;}$, is a particular solution of the Laplace equation, and using the identity

2.4 $$\frac{\partial v}{\partial N}\;ds=i\left(\frac{\partial v}{\partial \bar{z}}\;d\bar{z}+\frac{\partial v}{\partial z}\;dz\right),$$

equation (2.2) yields the global relation

2.5 $$\oint_{\partial \Omega }\;e^{-i\lambda z}\left[\frac{\partial u}{\partial N}+\lambda u\frac{dz}{ds}\right]ds=0,\;\lambda \in C^{\;}.$$

For the modified Helmholtz equation

2.6 $$u_{xx}+u_{yy}-k^{2}u=0,\;(x,y)\in \Omega ,$$

where *k*>0 is the wavenumber, we choose the particular solution $v=e^{\left(ik/2)(\bar{z}/\lambda -\lambda z\right)},$ $\lambda \in C^{\;}\setminus \{0\}$, and then equation (2.2) yields the global relation

2.7 $$\oint_{\partial \Omega }\;e^{\left(ik/2)[\bar{z}/\lambda -\lambda z\right]}\left[\frac{\partial u}{\partial N}+\frac{ku}{2}\left(\lambda \frac{dz}{ds}+\frac{1}{\lambda }\frac{d\bar{z}}{ds}\right)\right]ds=0.$$

For the Helmholtz equation

2.8 $$u_{xx}+u_{yy}+k^{2}u=0,\;(x,y)\in \Omega ,$$

we choose the particular solution $v=e^{\left(-ik/2)[\lambda z+\bar{z}/\lambda \right]}$, $\lambda \in C^{\;}\setminus \{0\},$ and then equation (2.2) yields the global relation

2.9 $$\oint_{\partial \Omega }\;e^{\left(-ik/2)[\lambda z+\bar{z}/\lambda \right]}\left[\frac{\partial u}{\partial N}+\frac{ku}{2}\left(\lambda \frac{dz}{ds}-\frac{1}{\lambda }\frac{d\bar{z}}{ds}\right)\right]ds=0.$$

For the Laplace, modified Helmholtz and Helmholtz equations with *u* real, a second global relation can be obtained from the global equations (2.5), (2.6), and (2.9) via Schwartz conjugation, i.e. by taking the complex conjugate of these equations and then replacing $\bar{\lambda }$ with λ.

### (a) A polygonal domain

In what follows, we consider the particular case where *Ω* is the interior of a polygon with *n* sides and corners ${\left\{z_{j}\right\}}_{1}^{n}$. The *j*th side of this polygon, which is the side between the edges *z*_*j*_ and *z*_*j*+1_, can be parametrized by the expression

2.10 $$z=m_{j}+th_{j},\;t\in [-1,1],$$

where *m*_*j*_ and *h*_*j*_ are defined by

2.11 $$m_{j}=\frac{z_{j}+z_{j+1}}{2},\;h_{j}=\frac{z_{j+1}-z_{j}}{2}.$$

Using the above parametrization in equation (2.5) and employing d*s*=|*h*| d*t*, we find that the global relation for the Laplace equation in a polygonal domain becomes

2.12 $$\sum_{j=1}^{n}e^{-im_{j}\lambda }\int_{-1}^{1}e^{-i\lambda h_{j}t}\left[|h_{j}|\frac{\partial u_{j}}{\partial N}+\lambda h_{j}u_{j}\right]dt=0,\;\lambda \in C^{\;}.$$

Typical boundary conditions for the *jth* side are given below:

2.13 $$\begin{array}{rl} \text{Dirichlet: }u_{j} & =g_{j}, \end{array}$$

2.14 $$\begin{array}{rl} \text{Neumann: }\frac{\partial u_{j}}{\partial N} & =g_{j} \end{array}$$

2.15 $$\begin{array}{rl} \mathrm{and}\;\text{Robin: }\frac{\partial u_{j}}{\partial N}+\gamma_{j}u_{j} & =g_{j}. \end{array}$$

In the above equations, *g*_*j*_ is a given function and *γ*_*j*_ is a given constant. If any of the above boundary conditions is prescribed on each side of the polygon, it follows that the global relation (2.12) involves *n* unknown functions. For the particular case that a Dirichlet boundary condition is prescribed on *every* side, it is rigorously shown in [6,7], that equation (2.12) together with the Schwartz conjugate of (2.12) uniquely determine the unknown values ${\left\{\partial u_{j}/\partial N\right\}}_{1}^{n}$.

In what follows, we present a simple procedure for computing numerically the *n* unknown boundary values for the Laplace, modified Helmholtz and Helmholtz equations in the case that any of the boundary conditions (2.13)–(2.15) is prescribed on the *j*th side of the polygon. We emphasize that it is possible to prescribe different types of boundary conditions on different sides.

## 3. The approximate global relation

We expand the functions ${\left\{u_{j}\right\}}_{1}^{n}$ and ${\left\{\partial u_{j}/\partial N\right\}}_{1}^{n}$ in terms of $N\in Z^{+}$ basis functions denoted by ${\left\{S_{l}(t)\right\}}_{0}^{N-1}$:

3.1 $$u_{j}(t)\approx \sum_{l=0}^{N-1}a_{l}^{j}S_{l}(t),\;\frac{\partial u_{j}}{\partial N}\approx \sum_{l=0}^{N-1}b_{l}^{j}S_{l}(t),\;j=1,\ldots ,n,\;t\in [-1,1],$$

where $a_{l}^{j}$ and $b_{l}^{j}$ are constants. Equations (2.13)–(2.15) imply that for each *j* there exists a linear relation between $a_{l}^{j}$ and $b_{l}^{j}$.

Let ${\hat{S}}_{l}(\lambda )$ denote the Fourier transform of $S_{l}(t)$, i.e.

3.2 $${\hat{S}}_{l}(\lambda )=\int_{-1}^{1}\;e^{-i\lambda t}S_{l}(t)\;dt,\;\lambda \in C^{\;}.$$

Substituting the expressions (3.1) into the global relation (2.12) and using equation (3.2), we find the following approximate global relation for the Laplace equation:

3.3 $$\sum_{s=1}^{n}\sum_{l=0}^{N-1}\;e^{-im_{s}\lambda }[|h_{s}|b_{l}^{s}+a_{l}^{s}\lambda h_{s}]{\hat{S}}_{l}(-i\lambda h_{s})=0,\;\lambda \in C^{\;}.$$

Similarly the approximate global relation for the modified Helmholtz equation is given by

3.4 $$\begin{array}{rl} & \sum_{s=1}^{n}\sum_{l=0}^{N}e^{\left(ik/2)[{\bar{m}}_{s}/\lambda -\lambda m_{s}\right]}\left[b_{l}^{s}|h_{s}|+a_{l}^{s}\frac{k}{2}\left(\lambda h_{s}+\frac{{\bar{h}}_{s}}{\lambda }\right)\right]{\hat{S}}_{l}\left[\frac{ik}{2}\left(\frac{{\bar{h}}_{s}}{\lambda }-\lambda h_{s}\right)\right]=0,\\ & \;\lambda \in C^{\;}\setminus \{0\}. \end{array}$$

Finally, the approximate global relation for Helmholtz equation is given by

3.5 $$\begin{array}{rl} & \sum_{s=1}^{n}\sum_{l=0}^{N}\;e^{\left(-ik/2)[{\bar{m}}_{s}/\lambda +\lambda m_{s}\right]}\left[b_{l}^{s}|h_{s}|+a_{l}^{s}\frac{k}{2}\left(\lambda h_{s}-\frac{{\bar{h}}_{s}}{\lambda }\right)\right]{\hat{S}}_{l}\left[-\frac{ik}{2}\left(\frac{{\bar{h}}_{s}}{\lambda }+\lambda h_{s}\right)\right]=0,\\ & \;\lambda \in C^{\;}\setminus \{0\}. \end{array}$$

Recalling that equation (2.12) implies a linear relation between $a_{l}^{j}$ and $b_{l}^{j}$, it follows that equation (3.3) together with its Schwartz conjugate are two equations with *nN* unknown constants. However, it is crucial to observe that λ is an *arbitrary* complex number. Thus by evaluating equation (3.3) and its Schwartz conjugate at the M points ${\left\{\lambda_{r}\right\}}_{1}^{M}$ where M≥nN/2, we can determine the unknown constants. Similar considerations are valid for the modified Helmholtz and Helmholtz equations.

### (a) The choice of the basis functions

Following Fornberg and co-worker [15], we let $S_{l}(t)=P_{l}(t)$, where *P*_*l*_(*t*) denotes the Legendre polynomials of order *l*. It is well known that the Fourier transform of *P*_*l*_(*t*) can be expressed in terms of the spherical Bessel function *J*_*l*_ [22], which in turn can be expressed in terms of the modified Bessel function *I*_*l*+1/2_. Also, an analytical expression for the Fourier transform of *P*_*l*_(*t*) is given in [23]. Thus, the expressions ${\hat{S}}_{l}$ which appear in (3.3)–(3.5) can be computed by

3.6 $$\int_{-1}^{1}e^{-i\lambda t}P_{l}(t)\;dt=J_{l}(\lambda )=\frac{\sqrt{2\pi \lambda }}{\lambda }I_{l+1/2}(\lambda )=2\sum_{k=0}^{l}\frac{(l+k)!}{(l-k)!k!}\left[\frac{{\left(-1\right)}^{l+k}\;e^{i\lambda }-e^{-i\lambda }}{{\left(2i\lambda \right)}^{k+1}}\right].$$

## 4. Collocation points

In order to motivate a convenient choice for the collocation points, we concentrate on the particular side with index *p*. The global relation (2.12) can be written in the form

4.1 $$\int_{-1}^{1}e^{-ih_{p}\lambda t}F_{p}(\lambda ,t)\;dt+\sum_{j=1,j\neq p}^{n}e^{i\lambda (m_{p}-m_{j})}\int_{-1}^{1}e^{-ih_{j}\lambda t}F_{j}(\lambda ,t)\;dt,\;p=1,\ldots ,n,$$

where *F*_*j*_(λ,*t*) is given by

4.2 $$F_{j}(\lambda ,t)=|h_{j}|\frac{\partial u_{j}}{\partial N}+\lambda h_{j}u_{j}.$$

Thus, for the particular side with index *p*, a natural choice for λ is *h*_*p*_λ=*real* number. However, it is also desirable to choose this real number in such a way that

4.3 $$R[i\lambda (m_{p}-m_{j})-ih_{j}\lambda t]<0,\;j=1,\ldots ,n,\;j\neq p,\;t\in [-1,1].$$

This condition guarantees that as λ→∞, the contribution from the other sides tends to zero. It is shown in [21] that for a *convex* polygon the condition (4.3) can indeed be satisfied provided that the above number is negative. Thus, for the side *p* we choose λ by

4.4 $$\lambda_{p}=-\frac{{\bar{h}}_{p}\tilde{f}}{|h_{p}{|}^{2}},\;\tilde{f}>0\;\text{or}\;\lambda_{p}=-{\bar{h}}_{p}f,\;f>0,\;p=1,\ldots ,n,$$

where $\tilde{f}$ is an arbitrary positive constant, and ${\bar{h}}_{p}$ denotes the complex conjugate of *h*_*p*_.

Similarly for the modified Helmholtz equation, the analogue of the first of equations (4.4) is the equation

4.5 kλp=−f~h¯p1+1+[k|hp|/f~]2|hp|2121+1+[k|hp|/f~]2|hp|2,f~>0, p=1,…,n,

which can also be written in the form of the second of equations (4.4). For the Helmholtz equation, the analogue of equation (4.5) is the equation

4.6 kλp=−f~h¯p1+1+[k|hp|/f~]2|hp|2121+1−[k|hp|/f~]2|hp|2,f~>0, p=1,…,n.

Thus, this can be written in the form of the second of equations (4.4) provided that $\tilde{f}>k|h_{p}|$. On the other hand, if $k|h_{p}|>\tilde{f}$, then

4.7 kλp=f~h¯p1+1+[k|hp|/f~]2|hp|212−1±i−1+[k|hp|/f~]2|hp|2,f~>0, p=1,…,n.

The above analysis shows that for a *convex* polygon an appropriate choice of the collocation points associated with the side *p* is $\lambda =-{\bar{h}}_{p}f$, *f*>0. We find it convenient to write *f* in the form below. Thus, associated with the side *p* we choose the following *M* collocation points

4.8 $$\lambda_{p,r}=-{\bar{h}}_{p}\frac{R}{M}r,\;R>0,\;M\in Z^{+},\;r=1,\ldots ,M.$$

In this way we choose *nM* collocation points, and hence by evaluating the two global relations at these points we obtain 2*nM* equations for *nN* unknowns. Hence, *M* must satisfy the constraint *M*≥*N*/2. Clearly, *M* specifies the ‘overdeterminedness’ of the linear system (the larger the *M* the larger the overdeterminedness). On the other hand, the parameter *R*/*M* defines the distance between two consecutive collocation points.

The error of approximating the Dirichlet and Neumann boundary values with the expressions (3.1) depends on *N*, whereas the condition number K of the relevant linear system depends on the *quadruple* (*R*,*n*,*N*,*M*). By scrutinizing a variety of numerical experiments, we have found the following necessary conditions for low K:

4.9 $$M\geq Nn,\;\frac{R}{M}\geq 2.$$

## 5. Numerical results

**Figure 1.**
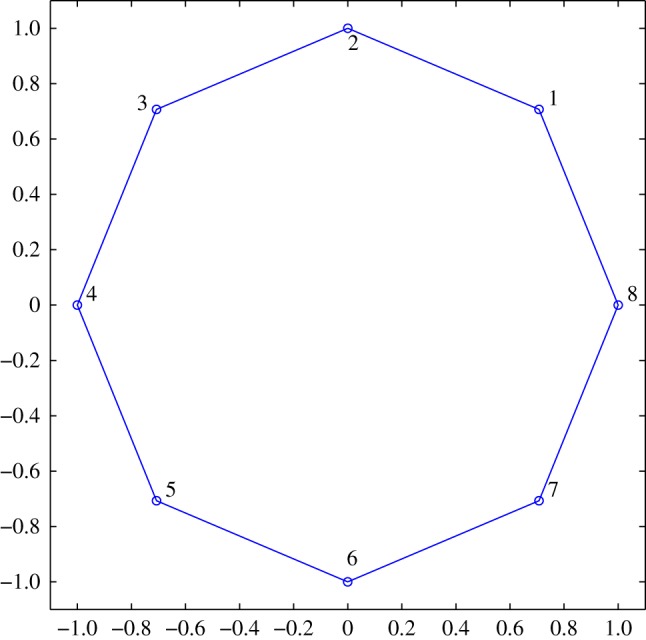
An octagon (typical polygon). The nodes are labelled counter clockwise. (Online version in colour.)

**Figure 2.**
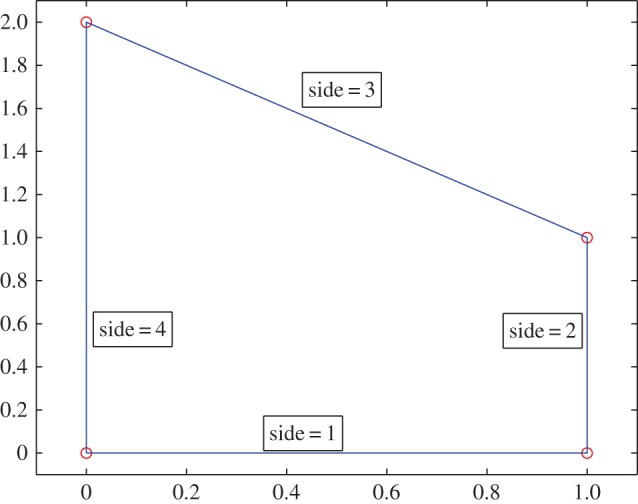
The trapezoidal geometry. (Online version in colour.)

In this section, we present the results of a parametric study of the key parameters in the unified transform, namely (*R*,*n*,*N*,*M*). Our chosen convex domains are the octagon shown in figure 1 and the trapezoid shown in figure 2. We compare the solution obtained via the unified transform with an analytical solution and the solution computed via the finite-element method.

### (a) The octagon geometry

For the octagon geometry shown in figure 1, we compare an analytical solution to the solution computed via the above approach. We denote the analytic solution by *u*^A^(*x*,*y*) and the solution obtained via the unified transform by *u*^UT^(*x*,*y*).

**Figure 3.**
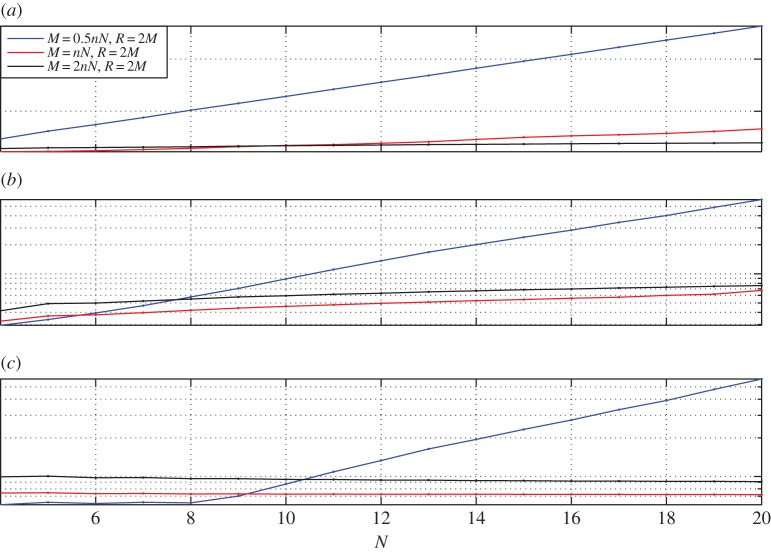
The condition number K for the octagon geometry. The blue solid line is *M*=*nN*/2, *R*=2*M*; the red solid line with dots is *M*=*nN*,*R*=2*M* and the solid black line with dots is *M*=2*nN*,*R*=2*M*. For the modified Helmholtz and Helmholtz equations $k=\sqrt{10}$. *N*, number of basis functions on each side. (*a*) Condition number for Laplace equation; (*b*) condition number for modified Helmholtz equation and (*c*) condition number for Helmholtz equation.

We choose the same analytical solution as the one used in [15]. The parametrization error for this particular solution has been studied in some detail by [15]. The condition number K versus the number of basis functions *N* on each side of the octagon for different choices of (*M*,*R*) is shown in figure 3. In the case of the Helmholtz and modified Helmholtz equations, we have fixed the wave number at $k=\sqrt{10}$. It can be observed in all three cases, namely the Laplace, the modified Helmholtz and the Helmholtz equations, the choice of *M*=*nN*, *R*/*M*=2 results in a low condition number.

**Figure 4.**
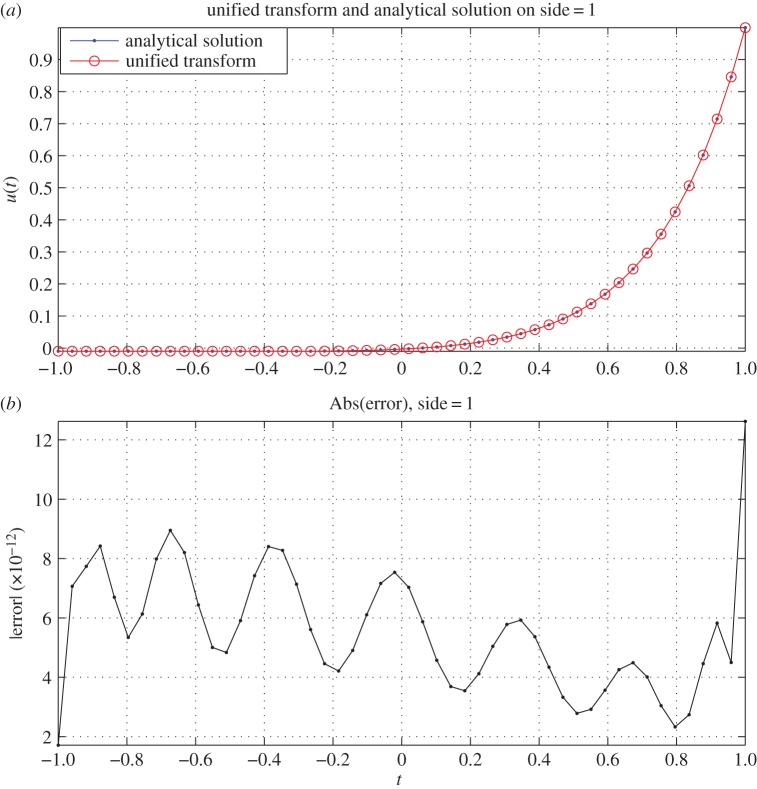
The solution and the corresponding absolute error on the first side (a typical side) of the octagon geometry for the modified Helmholtz equation. Subplot (*a*) shows the solution, where the blue solid line with dots is the analytical solution. The red dashed line with the circle is the unified transform solution. Subplot (*b*) shows the absolute error i.e. $|u_{1}^{A}(t)-u_{1}^{\mathrm{UT}}(t)|$. The wavenumber is $k=2\sqrt{26}$. The parameters associated with the unified transform solution are *n*=8, *N*=20, *M*=160 and *R*=320. The condition number of the system matrix is K=10.0315.

The comparison of the analytical solution and the solution obtained via the unified transform method for one side of the octagon is shown in figure 4. It can be observed that the unified transform provides an accurate solution. Furthermore, the choice of *M*=*nN*, *R*/*M*=2 results in the low condition number of K=10.0315.

### (b) The trapezoidal geometry

We now consider a case for which we cannot obtain an analytical solution. Consider the trapezoidal domain shown in figure 2. We choose a Robin boundary condition on side 1 and homogeneous Neumann boundary condition on the remaining sides:

5.1 $$\frac{\partial u_{1}}{\partial N}+u_{1}=1;\;\frac{\partial u_{j}}{\partial N}+u_{j}=0,\;j=2,3,4.$$

**Figure 5.**
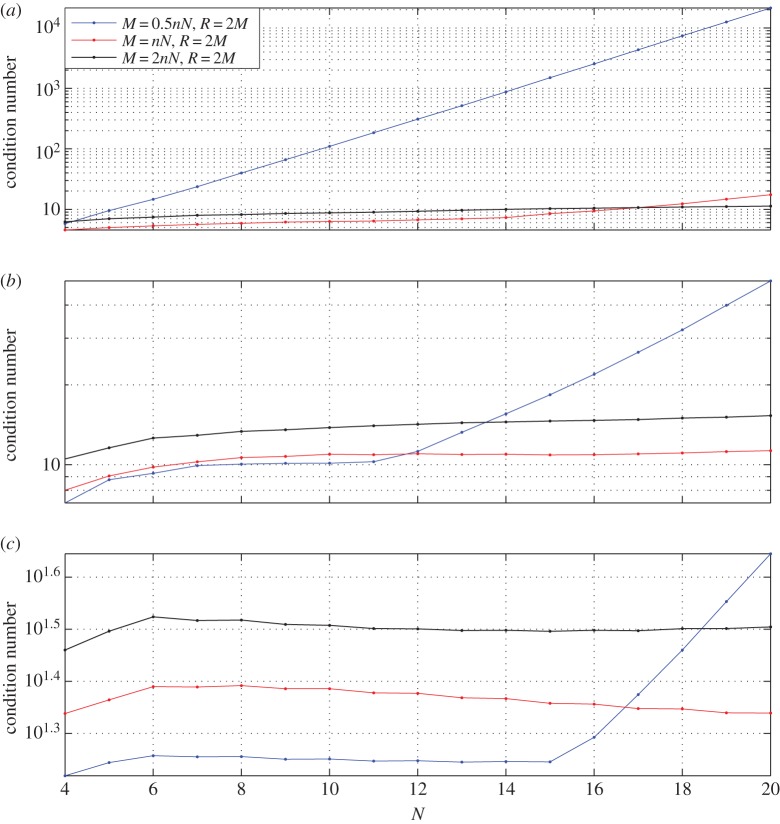
The condition number K for the trapezoidal geometry. The blue solid line is *M*=*nN*/2, *R*=2*M*; the red solid line with dots is *M*=*nN*, *R*=2*M* and the solid black line with dots is *M*=2*nN*, *R*=2*M*. For the modified Helmholtz and Helmholtz equations $k=\sqrt{10}.$ *N*, number of basis functions on each side. (*a*) Condition number for Laplace equation; (*b*) condition number for modified Helmholtz equation and (*c*) condition number for Helmholtz equation.

The results of the parametric study for the trapezoid is shown in figure 5. The results reconfirm the parametric study of the octagon and reveal that the choice *M*=*nN*, *R*/*M*=2 ensures a low condition number.

**Figure 6.**
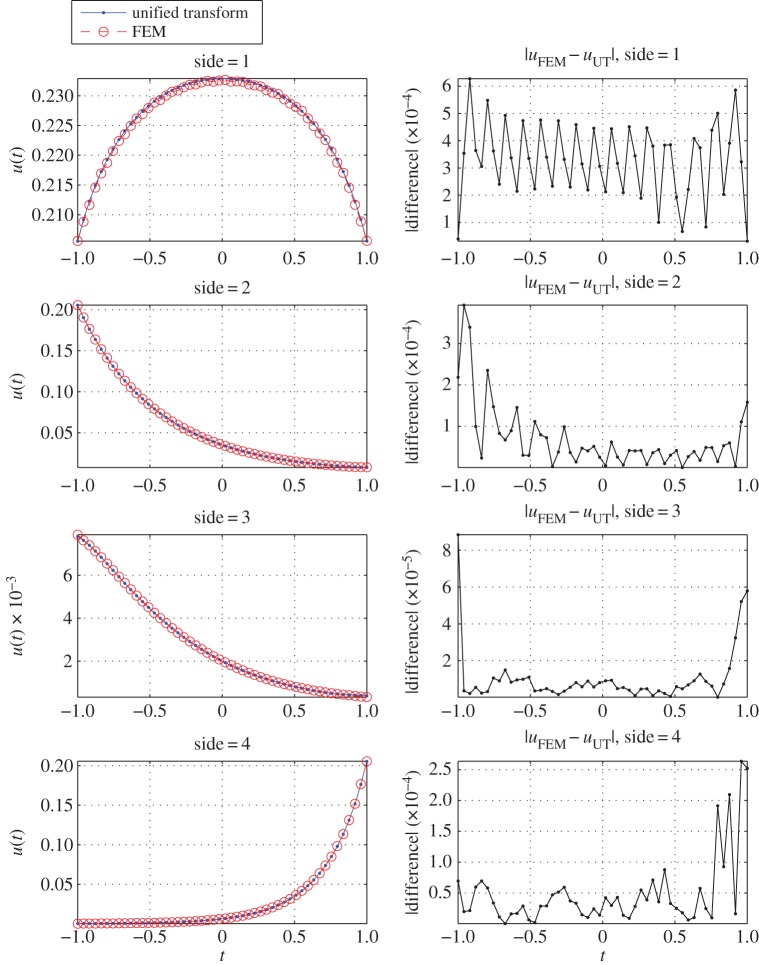
The solution *u*_*s*_(*t*) (*s*=1,…,4) on each side of the trapezoid for the modified Helmholtz equation. On the plots in the first column, the solid blue line with dots is the solution using the unified transform, whereas the red line with circles is the solution using the finite-element method. The figures on the second column show the corresponding absolute difference. The parameters associated with the unified transform are $N=10,\;M=4N,\;R=2M,\;\sqrt{k}=10$. Using these parameters the condition number of the system matrix is K=10.9499.

To validate the unified transform solution, we compare it with the solution obtained using the finite-element method [24]. We have used linear finite elements and the finite-element mesh of the trapezoidal geometry involves a total of 1954 nodes, 3715 triangles and the boundary has 191 edges. This comparison is shown in figure 6. The results show an excellent agreement between the finite element method and the unified transform.

## 6. Conclusion

The so-called *global relations* play a crucial role in the construction of analytical solutions for both evolution and elliptic PDEs. For the Laplace, modified Helmholtz and Helmholtz equations, the global relations are equations (2.5), (2.7) and (2.9), respectively, as well as the equations obtained from these equations by taking the complex conjugate and then replacing $\bar{\lambda }$ by λ. By employing the fact that λ is arbitrary, the global relations provide an elegant characterization of the Dirichlet to Neumann map.

It has been realized since the work of [16] that the global relations can be solved numerically. In this direction, different numerical techniques have been derived by several authors, see e.g. [13–21]. Here, following Fornberg and co-worker, we use the Legendre basis and also overdetermine the relevant system; furthermore motivated by the results of [21], we introduce a simple choice of ‘collocation’ points, see equation (4.8). This choice involves the positive number *R* and the positive integer *M*; *M* is a measure of overdeterminancy and *R*/*M* is the distance between two consecutive points.

We provide strong numerical evidence that if *M* and *R* satisfy the constraints given by equation (4.9) then the condition number of the associated system is of order 1. For example, for the trapezoidal domain analysed in [14,15] the condition number reduces from *O*(10^8^) to 4.7.

We next discuss the relation of the method presented in this paper with two other methods for solving linear elliptic PDEs in the literature which are also based on the relation (2.2):

(i) The *null field method* for the solution of the Helmholtz equation in the *exterior* of a bounded obstacle (originally introduced by Waterman in [25,26]) and (ii) a method for the solution of the Helmholtz equation above a periodic rough surface introduced by DeSanto [27] and further developed by DeSanto and co-workers [28–31]. The advantage of these two methods, as well as of the method described in this paper, is that they are boundary-based discretizations that do not involve the computation of singular integrals (as opposed to the discretizations of boundary integral equations). Relations between these methods are discussed in detail in [32], §10 and [33], §4. In the null-field method, *u* in (2.2) is the solution of the Helmholtz equation in the exterior of a bounded obstacle, and *v* is one of a countable family of separable solutions of the Helmholtz equation in polar coordinates that satisfies the appropriate radiation condition (see, e.g. [34], §7.5). The main difference between the null-field method and the method in this paper are the following: (i) the former method is used for an *exterior* of an obstacle, whereas the current method is used for the *interior* of a polygon and (ii) in the null-field method the unknown boundary value is expanded in a *global* basis (i.e. one in which the support of the basis functions is the whole of ∂*Ω*), whereas the method in this paper uses local bases (where each basis function is supported only on one side of the polygon). Regarding the method of DeSanto, *u* in (2.2) is the solution of the Helmholtz equation above a periodic rough surface, and *v* is chosen to be one of a countable family of separable solutions of the Helmholtz equation in Cartesian coordinates (so-called *generalized plane waves*) that satisfies the appropriate radiation condition. This method has been implemented with a variety of different bases chosen to express the unknown boundary value. Indeed, the authors of [30] proposed two different bases for the unknown boundary value to be expanded in: a local basis consisting of piecewise-constant basis functions, and a global basis consisting of the traces of the functions *v* in (2.2), and the authors of [31] proposed a variant of the second basis, where the complex-conjugates of the functions *v*, instead of the functions themselves, are used. An important point to note is that in both the null-field method and the method of DeSanto there is *no* flexibility in the choice of *v*, and thus there is *no* analogue of the question of how to choose λ in the global relations.

## Data accessibility

We have not used any data (measurements) collected by any organization including the University of Cambridge. This work involves a clear and rigorous description of a numerical implementation of the Fokas transform for solution of linear elliptic PDEs for convex polygons. The described method does not require or rely on any data.

## References

[1] Fokas AS 1997 A unified transform method for solving linear and certain nonlinear PDEs. Proc. R. Soc. Lond. A. 453, 1411–1443 (doi:10.1098/rspa.1997.0077)

[2] Fokas AS 2000 On the integrability of linear and nonlinear partial differential equations. J. Math. Phys. 41, 4188–4237 (doi:10.1063/1.533339)

[3] Fokas AS 2001 Two-dimensional linear PDEs in a convex polygon. Proc. R. Soc. Lond. A 457, 371–393 (doi:10.1098/rspa.2000.0671)

[4] Spence EA 2010 Boundary value problems for linear elliptic PDEs. PhD thesis, Cambridge, UK.

[5] Fokas AS Kalimeris K 2014 Eigenvalues for the Laplace operator in the interior of an equilateral triangle. Comput. Methods Funct. Theory 14, 1–33 (doi:10.1007/s40315-013-0038-7)

[6] Ashton ACL 2012 On the rigorous foundations of the Fokas method for linear elliptic partial differential equations. Proc. R. Soc. A. 468, 1325–1331 (doi:10.1098/rspa.2011.0478)

[7] Ashton ACL 2012 The spectral Dirichlet-Neumann map for Laplace's equation in a convex polygon. (http://arxiv.org/abs/1209.1584 [math.AP]).

[8] Ashton ACL Fokas AS 2013 Elliptic boundary value problems in convex polygons with low regularity boundary data via the unified transform. (http://arxiv.org/abs/1301.1490 [math.AP]).

[9] Ablowitz MJ Fokas AS Musslimani ZH 2006 On a new non-local formulation of water waves. J. Fluid Mech. 562, 313–343 (doi:10.1017/S0022112006001091)

[10] Ashton ACL Fokas AS 2011 A non-local formulation of rotational water waves. J. Fluid Mech. 689, 129–148 (doi:10.1017/jfm.2011.404)

[11] Fokas AS Nachbin A 2012 Water waves over a variable bottom: a non-local formulation and conformal mappings. J. Fluid Mech. 695, 288–309 (doi:10.1017/jfm.2012.19)

[12] Ambrose DM Nicholls DP 2014 Fokas integral equations for three dimensional layered-media scattering. J. Comput. Phys. 276, 1–25 (doi:10.1016/j.jcp.2014.07.018)

[13] Fokas AS Flyer N Smitheman SA Spence EA 2009 A semi-analytical numerical method for solving evolution and elliptic partial differential equations. J. Comput. Appl. Math. 227, 59–74 (doi:10.1016/j.cam.2008.07.036)

[14] Fornberg B Flyer N 2011 A numerical implementation of Fokas boundary integral approach: Laplace's equation on a polygonal domain. Proc. R. Soc. A 467, 2083–3003 (doi:10.1098/rspa.2011.0032)

[15] Davis CI Fornberg B 2014 A spectrally accurate numerical implementation of the Fokas transform method for Helmholtz-type PDEs. Complex Var. Elliptic Equ. 59, 564–577 (doi:10.1080/17476933.2013.766883)

[16] Fulton SR Fokas AS Xenophontos CA 2004 An analytical method for linear elliptic PDEs and it's numerical implementation. J. Comput. Appl. Math. 167, 465–483 (doi:10.1016/j.cam.2003.10.012)

[17] Saridakis YG Sifalakis AG Papadopoulou EP 2012 Efficient numerical solution of the generalized Dirichlet-Neumann map for linear elliptic PDEs in regular polygon domains. J. Comput. Appl. Math. 236, 2515–2528 (doi:10.1016/j.cam.2011.12.011)

[18] Sifalakis AG Fokas AS Fulton SR Saridakis YG 2008 The generalized Dirichlet-Neumann map for linear elliptic PDEs and its numerical implementation. J. Comput. Appl. Math. 219, 9–34 (doi:10.1016/j.cam.2007.07.012)

[19] Sifalakis AG Fulton SR Papadopoulou EP Saridakis YG 2009 Direct and iterative solution of the generalized Dirichlet-Neumann map for elliptic PDEs on square domains. J. Comput. Appl. Math. 227, 171–184 (doi:10.1016/j.cam.2008.07.025)

[20] Sifalakis AG Papadopoulou EP Saridakis YG 2007 Numerical study of iterative methods for the solution of the Dirichlet-Neumann map for linear elliptic PDEs on regular polygon domains. Int. J. Appl. Math. Comput. Sci. 4, 173–178.

[21] Smitheman SA Spence EA Fokas AS 2010 A spectral collocation method for the Laplace and modified Helmholtz equations in a convex polygon. IMA J. Numer. Anal. 30, 1184–1205 (doi:10.1093/imanum/drn079)

[22] Balanis CA 1989 Advanced engineering electromagnetics. New York, NY: Wiley.

[23] Fokas AS Smitheman SA 2014 The Fourier transforms of the Chebyshev and Legendre polynomials. Unified transform method for boundary value. (http://arxiv.org/abs/1211.4943 [math.NA]).

[24] Eriksson K Estep D Hansbo P Johnson C 2005 *Computational differential equations*. Lund, Sweden: Studentlitteratur.

[25] Waterman PC 1965 Matrix formulation of electromagnetic scattering. Proc. IEEE 53, 805–812 (doi:10.1109/PROC.1965.4058)

[26] Waterman PC 1969 New formulation of acoustic scattering. J. Acoust. Soc. Am. 45, 1417 (doi:10.1121/1.1911619)

[27] DeSanto JA 1981 Scattering from a perfectly reflecting arbitrary periodic surface: an exact theory. Radio Sci. 16, 1315–1326 (doi:10.1029/RS016i006p01315)

[28] DeSanto JA Erdmann G Hereman W Misra M 1998 Theoretical and computational aspects of scattering from rough surfaces: one-dimensional perfectly reflecting surfaces. Waves Random Media 8, 385–414 (doi:10.1088/0959-7174/8/4/001)

[29] DeSanto J.A Erdmann G Hereman W Misra M 2001 Theoretical and computational aspects of scattering from rough surfaces: one-dimensional transmitting interface. Waves Random Media 11, 425–454 (doi:10.1088/0959-7174/11/4/305)

[30] DeSanto JA Erdmann G Hereman W Krause B Misra M Swim E 2001 Theoretical and computational aspects of scattering from rough surfaces: two-dimensional perfectly reflecting surfaces using the spectral-coordinate method. Waves Random Media 11, 455–488 (doi:10.1088/0959-7174/11/4/306)

[31] Arens T Chandler-Wilde SN Desanto JA 2006 On integral equation and least squares methods for scattering by diffraction gratings. Commun. Comput. Phys. 1, 1010–1042.

[32] Spence EA In press When all else fails, integrate by parts-an overview of new and old variational formulations for linear elliptic PDEs. In *Unified transform method for boundary value problems: applications and advances*. Philadelphia, PA: SIAM.

[33] Chandler-Wilde SN Langdon S In press Acoustic scattering: high frequency boundary element methods and the unified transform methods. In *Unified transform method for boundary value problems: applications and advances*. Philadelphia, PA: SIAM.

[34] Martin PA 2006 Multiple scattering: interaction of time-harmonic waves with *N* obstacles. Cambridge, UK: Cambridge University Press.

